# Plant Cell Wall Integrity Perturbations and Priming for Defense

**DOI:** 10.3390/plants11243539

**Published:** 2022-12-15

**Authors:** Sivakumar Swaminathan, Vincenzo Lionetti, Olga A. Zabotina

**Affiliations:** 1 Roy J. Carver Department of Biochemistry, Biophysics and Molecular Biology, Iowa State University, Ames, IA 50011, USA; 2 Dipartimento di Biologia e Biotecnologie “Charles Darwin”, Sapienza Università di Roma, 00185 Rome, Italy

**Keywords:** plant cell wall, polysaccharides, cell wall integrity (CWI), cell-wall-modifying enzymes (CWMEs), cell-wall-digesting enzymes (CWDEs), damage-associated molecular patterns (DAMPs), surveillance, signaling cascade, pattern triggered immunity

## Abstract

A plant cell wall is a highly complex structure consisting of networks of polysaccharides, proteins, and polyphenols that dynamically change during growth and development in various tissues. The cell wall not only acts as a physical barrier but also dynamically responds to disturbances caused by biotic and abiotic stresses. Plants have well-established surveillance mechanisms to detect any cell wall perturbations. Specific immune signaling pathways are triggered to contrast biotic or abiotic forces, including cascades dedicated to reinforcing the cell wall structure. This review summarizes the recent developments in molecular mechanisms underlying maintenance of cell wall integrity in plant–pathogen and parasitic interactions. Subjects such as the effect of altered expression of endogenous plant cell-wall-related genes or apoplastic expression of microbial cell-wall-modifying enzymes on cell wall integrity are covered. Targeted genetic modifications as a tool to study the potential of cell wall elicitors, priming of signaling pathways, and the outcome of disease resistance phenotypes are also discussed. The prime importance of understanding the intricate details and complete picture of plant immunity emerges, ultimately to engineer new strategies to improve crop productivity and sustainability.

## 1. Introduction 

### 1.1. Plant Cell Wall Components

Plant cell walls (CWs) show highly heterogeneous structures across different species, tissues, and developmental stages. Plant CWs are complex and dynamic biological networks composed of interacting polysaccharides, proteins, phenolic compounds, minerals, and water. CWs provide support and protection to plants, determine their morphology, and mediate cell adhesion and cell-to-cell communication during growth and development [1].

The CW polysaccharides range from linear to highly branched polymers. Cellulose, the main component of all types of CWs, possesses high tensile strength. It is composed of multiple hydrogen-bonded β-1,4-linked glucan chains to form cable-like microfibrils [2]. Hemicelluloses comprise different classes of polysaccharides (xyloglucans, xylans, and mannans) with sugar backbones linked by equatorial β-1,4 linkages, some of which are decorated with side chains [3]. Xyloglucan, the most abundant hemicellulose in type I primary walls, inherits a β-1,4-linked glucan backbone decorated with side chains consisting of xylose, galactose, and fucose residues. Xylans are the predominant hemicellulose in type II primary walls and eudicot secondary walls but are also present in small amounts in type I primary walls. Xylans have β-1,4-linked xylosyl backbones with side chains consisting of arabinose and glucuronic acid, some of which are methylated or feruloylated. Mannans, prevalent in gymnosperms and also present in other plant taxa, are polymers of β-1,4-linked mannose. Glucomannans consist of repeating disaccharide subunits of glucose and mannose joined by β-1,4 linkages. Mixed-linkage glucans (MLGs), common in rapidly growing grass tissues, contain β-1,4-linked stretches of glucose interspersed with β-1,3 linkages [3].

Pectins (homogalacturonan, xylogalacturonan, rhamnogalacturonan I, and rhamnogalacturonan II) are acidic polysaccharides enriched in galacturonic acid residues. Homogalacturonan (HG), the most abundant pectin, consists of continuous α-1,4-linked galacturonic acid residues that can be methylesterified at the C6 carboxyl groups and acetylated at O2 and O3 positions [4]. The backbone of rhamnogalacturonan I (RG I) consists of alternating galacturonic acid and rhamnose residues decorated with arabinan, galactan, and arabinogalactan side chains. Rhamnogalacturonan II (RG II), a highly conserved and complex pectin, consists of HG backbone decorated with side chains containing 13 different sugar subunits and over 20 distinct glycosyl linkages. Hemicelluloses and pectins are matrix polysaccharides. The nature of the structural diversity of pectin is due to its complex biosynthetic process, which requires a minimum of 67 different transferases, including glycosyltransferases, methyltransferases, and acetyltransferases [5,6].

Enzymes and nonenzymatic proteins are also present in CWs. Enzymes, including glycosyl-hydrolases, oxidoreductases, lyases, and esterases, are mainly involved in CW remodeling during different growth and defense processes. Structural proteins present in the CW include extensins, proline-rich proteins, and arabinogalactan proteins [1]. Lignin, abundant in secondary walls, is a hydrophobic, polyphenolic compound made of covalently linked monolignol subunits that undergo redox-mediated polymerization. Lignin can be covalently linked to the ferulate side chains of xylans [7].

### 1.2. Overview of CW Integrity Systems Involved in Plant Biotic Stress 

A CW has an established role in maintaining and determining cell shape, resisting internal turgor pressure, directing cell and plant growth, contributing to plant morphology, and regulating diffusion through the apoplast. Plants continually face environmental and biotic stresses, and these stressful conditions force the plants to evolve and develop monitoring systems to deal with the harsh conditions [8,9,10]. Although previously considered only as a passive barrier against pathogens, it is now clear that the CW is a dynamic structure and the main site harboring different plant monitoring systems for perception and signaling plant immunity. 

The molecular mechanisms underlying CW integrity (CWI) maintenance, aimed to monitor and fine tune a CW’s structural and functional integrity, are of particular interest. The mechanism involves various plasma membrane receptors and complex signal transduction pathways, which help the plant to maintain its growth through development as well as manage different adverse abiotic and biotic stresses [11,12,13]. ‘Plant-self’-derived damage-associated molecular patterns (DAMPs) comprise plant molecules released in the apoplast. The DAMPs include wall-derived glycans and peptides, which, upon exposure of the plant to different stresses, are either de novo synthesized or processed to produce mature active ligands. Plants have a dedicated innate immunity system to monitor and maintain CWI, which comprises a diverse set of plasma-membrane-resident pattern recognition receptors (PRRs) to detect the DAMPs. Plants reinforce the perception system by using PRRs dedicated to detecting ‘non-self’-microbe-associated molecular patterns (MAMPs) or herbivore-associated molecular patterns (HAMPs) and activate pattern-triggered immunity (PTI). MAMPs are highly conserved microbial molecules derived from pathogens or parasites. The well-known MAMPs are flg22 from bacterial flagellin, chitooligomers from fungal CWs, and exoskeletons of insects [14]. The plant immune system can also recognize microbial effectors (Avr proteins) through cytoplasmic resistance (R) proteins, which results in triggering effector-triggered immunity (ETI) [15,16]. 

**Figure 1:**
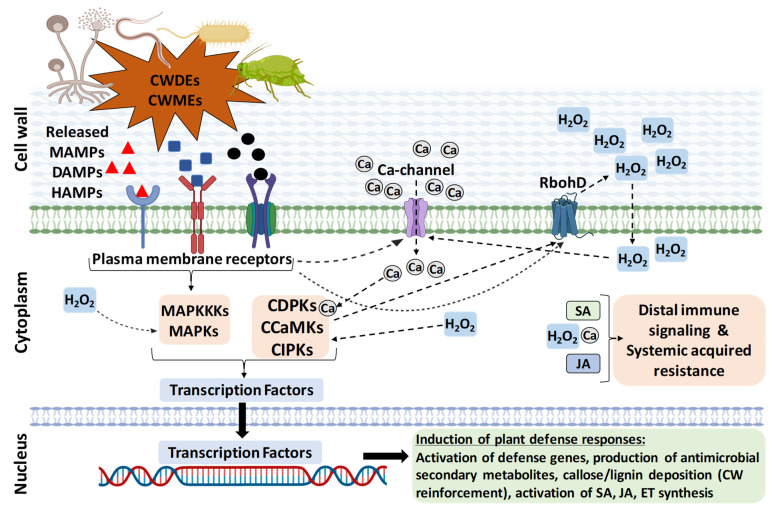
An overview of plant defense pathway triggered by perturbation of cell wall integrity during biotic stresses. The DAMPs, MAMPs, or HAMPs elicitors released due to biotic stresses are sensed through the plasma-membrane-localized pattern-recognition receptors (PRR) that activate the host defense pathway. The elicitor binding to PRRs activates a series of events, such as activation of Ca-channels and Ca influx, activation of reactive oxygen species production (ROS burst; H_2_O_2_), and protein kinases (MAPKs) cascade activation, and these are inter-linked with each other. The MAPK and Ca-dependent kinase (CDPKs, CCaMKs, and CIPKs) cascades further activate transcriptional reprogramming, defense-related PTI genes, reinforcement of cell wall (callose and lignin deposition), generation of anti-microbial secondary metabolites, and activation of ET, SA, and JA hormone synthesis. Locally activated immunity is amplified through these hormones. Immunity may also involve the spread of defense responses to distal tissues, resulting in systemic acquired resistance (SAR). It should be emphasized that a general view of only the pattern triggered immunity is described here and that each pathosystem may involve specific molecular interactions.

According to the “zig-zag model”, PTI is considered as the first line of defense activated by MAMPs through specific PRR [12]. ETI is the second layer of defense, triggered by the secreted microbial genotype-specific pathogenicity effectors and recognized by plant genotype-specific receptor proteins (R). The effects of PTI include rapid activation of a broad spectrum of defense responses, such as oxidative burst, Ca_2_+ influx, nitric oxide accumulation, protein kinases activation, and cell wall reinforcement (Figure 1). Transcriptional reprogramming leads to induction of defense-related genes to generate secondary metabolites/anti-microbial compounds, induction of enzymes to digest the microbial CW (chitinases, β-1-3 glucanases), and activation of the ethylene (ET), salicylic acid (SA), and jasmonic acid (JA) pathways, which are late outcomes of the PTI response [17,18] (Figure 1). ETI is characterized by a higher and longer response, which, in many cases, results in a localized programmed cell death known as a “hypersensitive response” [19]. PTI and ETI are inter-dependent and mutually enhance each other to provide total immunity to plants, which might share signaling components and responses produced by DAMPs [20,21,22]. Moreover, local activation of immunity confers wide-spectrum protection against pathogens in distal tissues, known as systemic acquired resistance (SAR), which mainly involves the hormone SA and JA [13].

Research findings extensively demonstrated that alterations in CWI can be achieved either by intentional chemical perturbation, overexpression (OE), or mutation of CW-related genes/enzymes. As a result, the triggered signaling pathways can significantly impact disease resistance and/or abiotic stresses. CW danger signaling can also be stimulated by expressing microbial CW-degrading enzymes (CWDEs) in planta. Fungi produce a notable quantity and variety of CWDEs, mostly belonging to glycosyl hydrolases [23]. Soft rot bacteria also synthesize significant CWDEs that contribute to their virulence [24]. Several parasitic nematodes and phytophagous insects also produce CWDEs for their invasion [25,26,27]. Moreover, parasitic plants produce a haustorium highly expressing CWDEs [28]. A specific CWDE is used to achieve desired modification in the CW components and is a highly valuable tool to understand the physiological consequences arising from that particular modification [29]. 

It was initially thought that the disease resistance phenotypes observed with alterations in CWI were due to the inability of mis-adapted pathogens to overcome the genetically modified wall compositions/structures in the genetically modified plants [11]. However, later studies found that a CW is a highly dynamic component of a cell and not just a passive barrier. CW alterations and DAMPs trigger complex defensive signaling immune pathways to fight against plant pathogens, inducing defense responses and reinforcement of the CW [11]. Recent research with a large set of Arabidopsis CW mutants revealed that CWs of mutant plants exhibited high diversity of composition alterations, as revealed by glycome profiling [30]. Moreover, it reported that plant CWs are determinants of immune responses and illustrated the relevance of CW composition in determining disease-resistance phenotypes to pathogens with different parasitic styles.

CWI alterations, in some instances, can provide tolerance/resistance to pathogens, while, in other cases, they result in susceptibility. Therefore, expression of endogenous CW-modifying enzymes (CWMEs) or microbial CWDEs and their inhibitors in planta is a highly useful tool mainly to investigate the molecular mechanisms behind CWI maintenance during stress, unearth plant immunity and complex signaling pathways, and host plant–microbe interactions. Further, it is also useful in improving crop protection against plant pathogens [29,31,32,33,34,35]. 

The artificial CW modifications could induce plant defense immunity reactions constitutively even prior to pathogen attack, and these defenses may be able to help the plants to resist the pathogen during actual attack [35,36]. Several efforts were made to unravel the complexity behind the CW role in plant pathogen resistance. Indeed, increasing evidence demonstrates that changes in CW composition, either via altering polysaccharides biosynthesis or post-synthetic modifications of polysaccharides *in muro*, can induce reactions similar to those induced during plant responses to stresses. The research findings also indicated that studies on CW modifications and defense priming are highly complicated and not yet fully elucidated in many cases. There is much to investigate to completely understand the complex molecular nature of the host plant–pathogen interaction [11,37].

This review is mainly focused on the recent advances in molecular mechanisms underlying CWI maintenance in plant–pathogen and parasitic interactions. The CWI perturbations by herbivory and wounding are not covered in our review since these topics were recently reviewed in several papers [25,26,27]. Here, we reviewed primarily the studies related to the CW alterations generated by mutations or expression of plant CW synthesizing enzymes, CWMEs, or microbial CWDEs and their effect on priming of signaling cascades and disease resistance phenotypes.

## 2. Effect of Alterations of CWI on Plant Immunity and Pathogen Resistance

### 2.1. CW Cellulose Integrity and Plant Immunity

Cellulose is the main component of the primary and secondary CW and confers strength to these structures. It is synthesized at the plasma membrane by the catalytic subunits of the cellulose synthase (CESA) protein complex. Altering or mutating the expression of *CESA* genes has a specific impact on CW impairment and results in release of DAMPs and activation of immune signaling, which further results in either pathogen susceptibility or resistance [11]. Arabidopsis *irregular xylem* (*irx*) *5/3/1* CW mutants, defective in *CESA* subunits (*CESA4/7/8*) required for secondary CW formation, displayed enhanced resistance to several pathogens, including the necrotrophic fungi *Plectosphaerella cucumerina* and *Botrytis cinerea*, the vascular bacterium *Ralstonia solanacearum,* and the vascular fungus *Fusarium oxysporum* [38,39]. The disease resistance development was due to constitutive activation of plant immune responses, accounted mainly by the abscisic acid (ABA)-responsive signaling pathway and not due to SA, JA, and ET pathways. The defense pathway elements induced were pathogen-responsive (PR) genes, transcriptional regulators (e.g., *ATR1*), enzymes involved in synthesis and activation of antimicrobial secondary metabolites (e.g., CYP79B2 and CYP79B3), and antimicrobial peptides belonging to four distinct families (LTPs, thionins [THs], snakin/GASA, and pEARLY). Impairment of ABA signaling in the Arabidopsis ABA mutants (*aba1-6*) resulted in reduced cellulose and increased uronic acid in its CW, which further resulted in resistance to *P. cucumerina*. It reveals that the ABA pathway negatively regulates defense mechanism [40]. 

Similarly, mutating an Arabidopsis *MYB46* transcription factor that regulates the expression of *CESA4/7/8* resulted in enhanced resistance to *B. cinerea* [41]. The enhanced resistance was due to heightened activation of the JA-regulated plant defensin gene *PDF1.2a*, which is effective against necrotrophic fungal pathogens and enhanced induction of peroxidase genes. Altered impairment of cellulose synthesis in the primary CW also leads to altered disease resistance. For example, CESA3-defective *isoxaben resistant* (*ixr1*)/*constitutive expression of VSP* (*cev1*) mutant plants, having constitutively activated ET and JA signaling genes *THI2.1* and *PDF1.2*, respectively, were more resistant to *B. cinerea*, *Psuedomonas syringae*, whereas their resistance to *R. solanacearum* and *P. cucumerina* was similar to wild type plants [38,42]. 

**Figure 2:**
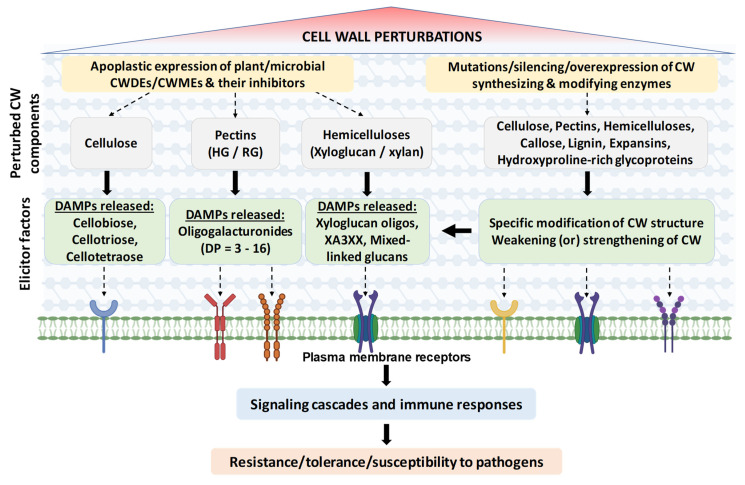
Induced CW perturbations and related signaling. A targeted modification of the CW structures (polysaccharides, phenolics, and proteins) can be achieved by apoplastic overexpression/mutation/silencing the plant endogenous CW synthesizing enzymes, CWMEs, and their inhibitors. As an alternative strategy, exogenous microbial CWDEs can be expressed in the apoplast as well. This results in a different extent of CW perturbations that can result in production of a plethora of DAMPs. DAMPs are perceived by specific receptors to trigger single or multiple defense signaling pathways, which results in different levels of immunity and tolerance/resistance to pathogens. Targeted modification of CW components in planta represents a useful tool to learn about the molecular mechanisms behind CWI maintenance during stress. This knowledge can be exploited to improve crop protection against pathogens.

In tomatoes, silencing endo- β-1,4-glucanase family proteins KORRIGAN1, Cel1, and Cel2 resulted in increased resistance against necrotrophic pathogen *B. cinerea* due to faster and enhanced accumulation of callose upon infection. Increased transcript levels of *lipoxygenase2* (*LOX2*) and *PDF1.2* and increased JA level were noticed in the silenced plants. However, silencing of these genes benefited colonization of the bacterial biotroph *P. syringae* pv *tomato* DC3000. Accordingly, the silencing of barley *Cellulose Synthase Like D2* enhanced susceptibility to powdery mildew biotrophic fungus *Blumeria graminis* [43]. Here, the increase in susceptibility was associated with lower cellulose contents in epidermal CWs and increased digestion by fungal CWDEs. This evidence suggests that impairment of cellulose synthesis is a negative factor for necrotrophs but a susceptibility factor against biotrophs [44,45] (Figure 2). 

### 2.2. CW Pectin Integrity and Plant Immunity

Pectins have several important functions, such as promoting cell-to-cell adhesion, providing structural support in primary CW, and influencing secondary CW formation, mainly in fibers and woody tissues [46,47,48]. 

In Arabidopsis, glucuronate 4-epimerases (GAEs) are involved in synthesizing nucleotide sugar donors for pectin biosynthesis. Studies showed that impairment of Arabidopsis *glucuronate 4-epimerases* (*GAEs*) results in susceptibility towards *P. syringae* and *B. cinerea* [49]. Further, *gae1gae6* double-mutant plants’ CWs had reduced pectin, specifically HG and likely RG I. In particular, *gae1gae6* double-mutant plants were more susceptible to both pathogens and were hyper-responsive to the JA, emphasizing a link between pectin-mediated plant defense and JA signaling [49].

Functional analyses showed that *AtERF014*, a nuclear-localized transcriptional activator, plays dual regulation in pectin biosynthesis and Arabidopsis immunity to two different pathogens. A study showed that overexpression of *AtERF014* increased Arabidopsis resistance against *Pst* DC3000 but decreased the resistance to *B. cinerea* [50]. Overexpression and silencing of *AtERF014* increased and decreased pectin content in the CW, respectively. The transcript level of the SA pathway (*AtPR1* and *AtPR5*) and JA pathway (*AtPDF1.2*) genes increased in overexpressor transgenic plants upon infection with these two pathogens. Opposite results were observed in *AtERF014-RNAi* lines [50].

Interestingly, a study reported that host Arabidopsis PMR6 (encodes a glycosylphosphatidylinositol-anchored pectate lyase-like protein) and PMR5 (an acetylation protein) were required for successful establishment of powdery mildew pathogens *Erysiphe cichoracearum* and *C. higginsianum*. Arabidopsis mutants *pmr5* and *pmr6* were powdery-mildew-resistant but were more susceptible to *P. syringae* and *B. cinerea* [51,52,53]. These mutants possessed pectin-enriched CWs with less esterification, and *pmr5* and *pmr6* resistance represents a novel form of disease resistance that does not require pathways such as SA and JA-ET. The synthesis of pectins and xylans was reduced in Arabidopsis starch-deficient mutants, *phosphoglucomutase* (*pgm*), and, accordingly, these mutants were impaired in penetration resistance to *C. higginsianum* [54]. 

Pectin is synthesized in a highly methylesterified form in the Golgi cisternae and partially de-methylesterified at apoplast by pectin methylesterases (PMEs). PMEs activity is post-transcriptionally regulated by PME inhibitors (PMEIs). Arabidopsis produces a local and strong induction of PME activity during infection of *B. cinerea* and *P. syringae* [55,56]. PME overexpression in strawberry can result in increased resistance to *B. cinerea* due to accumulation of oligogalacturonides (OGs) and primed immunity [57]. *Atpme17* mutants were highly susceptible to *B. cinerea* owing to down-regulation of the JA-ET pathway marker gene, *PDF1.2*, in spite of the induction of callose and reactive oxygen species (ROS). Similar results were also observed in other plant–pathogen interactions [57,58,59,60]. PME-mediated de-methylesterification of pectin can also generate methanol, an alarm signal that also causes priming of defense in nearby plants [61]. 

PMEs are also involved in regulating the pectin methylesterification status that strongly influences resistance to pathogens [31]. Highly methylesterified pectin results in good tolerance to the CWDEs secreted by pathogens. For instance, the degree of methylesterification in potato cultivars is positively correlated with their resistance to *Pectobacterium carotovorum* [62]. PME activity and pectin methylesterification are dynamically modulated by PMEIs during *B. cinerea* infection, pointing to AtPMEI10, AtPMEI11, and AtPMEI12 as mediators of the maintenance of CWI in plant immunity [32,63]. Arabidopsis immunity to *B. cinerea* was compromised in *pmei10*, *pmei11*, and *pmei12* mutants due to a lower amount of pectin methylesterification during infection, whereas the apoplastic overexpression of two PME inhibitors, *AtPMEI-1* and *AtPMEI-2,* increased the pectin methylesterification and increased the resistance to *B. cinerea* and *P. carotovorum* [33,64], and similar results were observed with a kiwi PMEI [65].

PME can also be the target of movement proteins produced by viruses for easy movement from cell to cell in plant tissues [56,66]. Notably, tobacco plants constitutively expressing a *PMEI* from *Actinidia chinensis* (*AcPMEI*) at apoplast resulted in reduced systemic movement of *Tobacco mosaic virus* (TMV). Comparable results were noticed in Arabidopsis with *AtPMEI-2* against *Turnip vein clearing virus* [67]. PMEI can also act as antimicrobial proteins against microbes, which was observed in *Capsicum annuam* expressing CaPMEI1 against *F. oxysporum*, *Alternaria brassicicola*, *X. campestris pv. vesicatoria*, *P. syringae pv. tomato,* and *B. cinerea* [68]. In cotton (*Gossypium hirsutum*), the ectopic expression of *GhPMEI3* inhibited the activity of GhPME2/GhPME31 and repressed the expression of a fungal polygalacturonase (PG) encoding gene, *VdPG1*, which resulted in increased resistance to *Verticillium dahliae* [69].

Plants overexpressing *Aspergillus niger* PME (AnPME) at apoplast showed a 50% reduction in methylester content, increased arabinose content, and decreased galacturonic acid content and a severe dwarf phenotype [70]. *AnPME* plants became insensitive to osmotic stress, and their susceptibility to *B. cinerea* was similar to wild type plants. Despite their compromised CWs, the lack of pathogen susceptibility might be due to the induction of many defense response genes.

Another important modification of pectin is acetylation, which occurs during its exocytosis and incorporation into the CW [3,71]. Two enzymes modulate the acetylation degree of pectin. Pectin acetyltransferases transfer acetyl residues to polysaccharides, while pectin acetylesterase (PAE) cleaves acetyl groups from pectin [72,73,74]. A recent study showed that overexpressing an endogenous pectin acetylesterase, *CsPAE2*, in apoplast makes *Citrus sinensis* become more resistant against bacterial canker disease, and susceptibility was observed in the mutants [75]. 

An earlier study also reported reduced pectin/xyloglucan CW acetylation in Arabidopsis and *Brachypodium distachyon* by apoplastic overexpression of an *A. nidulans* (AnRAE) acetylesterase, which led to enhanced resistance against *B. cinerea* and *Bipolaris sorokiniana*, respectively [36]. In *AnRAE*-overexpressing transgenic plants, the deacetylated weakened CW was easily digested by glycosyl hydrolases, which further resulted in generation of DAMPs and constitutive priming of immune pathway genes (Figure 2). 

Polygalacturonases (PG) are glycosyl hydrolases that depolymerize the pectic HG [76]. These enzymes are present endogenously in plants and also present in microbes and insects [77]. An Arabidopsis *ADPG2* negatively regulates disease resistance, and its knockdown mutants and overexpression lines exhibited decreased and increased pectin degradation of CW. This resulted in decreased (in spite of induced *PR5* level) and increased resistance to *P. syringae*, respectively [78]. Similarly, Arabidopsis plants expressing a fungal, *A. niger*, *AnPG* were found highly resistant to *B. cinerea* [79]. One of the adaptive defensive responses of plants to microbial and insect PGs is the synthesis of PG-inhibiting proteins (PGIPs) [80]. In agreement with that, overexpression of some of the PGIPs in different plant species resulted in improved resistance to different necrotrophic fungi and bacteria [81,82]. 

Alterations in host plant pectins integrity in CW were also caused by CWDEs of nematodes. Transgenic Arabidopsis expressing a *Heterodera schachtii* cellulose binding secretory protein (*HsCBP*) gene showed that HsCBP directly interacts with PME3 to reduce the level of methylesterification to aid in cyst nematode parasitism. As a result, the transgenic plants developed longer roots and exhibited higher susceptibility to *H. schachtii* [83]. The same study showed that transgenic plants overexpressing *AtPME3* had longer roots and increased susceptibility to *H. schachtii*, and opposite results were observed in knockout mutants. 

Interestingly, transgenic Arabidopsis overexpressing a plant *β-1,3-endoglucanase* (*At4g16260*) exhibited a reduction in *H. schachtii* infection [84]. The study suggested that the host plant β-1,3-endoglucanase has a potential role in the defense response by suppressing the detrimental effect caused by nematode effector protein and by hydrolyzing the fungal CW β-1,3-glucan. Opposite results were observed with the mutant *At4g16260* plants. A study with a novel pectate lyase gene *Mg-PEL1* from *Meloidogyne graminicola*, transiently expressed in *Nicotiana benthamiana,* demonstrated that CW localization of Mg-PEL1 was required to activate plant defense response genes (*PR5, PAL, NPR1*), programmed plant cell death, and ROS accumulation [85]. 

### 2.3. CW Hemicellulose Integrity and Plant Immunity

Alteration of wall xylose content, the moiety present in xylans and xyloglucans, affects resistance of plants to pathogens. Transgenic barley plants co-expressing two glycosyltransferase (GT)-encoding genes (*GT43* and *GT47*) responsible for xylan backbone biosynthesis resulted in increased accumulation of heteroxylan in the papillae that resulted in penetration resistance against *B. graminis* [86]. In contrast, Arabidopsis *er* plants (impaired in ERECTA receptor-like kinase) and *agb1* and *agg1agg2* mutants (impaired heterotrimeric G proteins) had heavily reduced xylose content [87]. These mutants were hyper-susceptible to necrotrophic fungus *P. cucumerina* despite the induction of JA, SA, ET, and ABA signal transduction pathway genes. 

In the Arabidopsis *det3* (*de-etiolated3*) and *irx6-1* (*irregular xylem 6-1*) mutants, a higher accumulation of xylose content in the CW was observed, and these mutants were highly resistant to *P. cucumerina* [87,88,89]. In nature, for recycling xyloglucan during plant metabolism and growth, xylose side chains must be removed first for proper degradation of the xyloglucan backbone later, and α-xylosidase activity is necessary to remove the xylose subunits. A study showed that an Arabidopsis *xyl1-2* mutant lacking a α- xylosidase had modified xyloglucan in the CW and exhibited higher resistance to *P. cucumerina* [87,90].

It is interesting to note that an Arabidopsis BETA-XYLOSIDASE4 (BXL4) protein with xylosidase/arabinosidase bifunctional activity was induced in apoplast as a defense response upon infection with *B. cinerea* [91]. The study reported that the *bxl4* mutants were susceptible to *B. cinerea*, while resistance increased in overexpression lines. Ectopic apoplast expression of *AtBXL4* in Arabidopsis seed coat epidermal cells rescued the *bxl1* mutant phenotype. The results concluded that BXL4 is a xylosidase/arabinosidase, its expression is upregulated under pathogen attack, and the enzyme is secreted to the apoplast. At apoplast, the enzymatic removal of arabinose/xylose side-chains in the primary CW polysaccharides results in generation of DAMPs, priming of the defense pathway genes, and eventually contributes to resistance against *B. cinerea* [91]. In another study, apoplastic expression of *F. graminearum* xylanase (that degrades xylan) in tobacco and Arabidopsis resulted in enhancing the immunity against *P. syringae* pv. *tabaci* or pv. *Maculicola,* and prolonged *PR1* expression was detected after *P. syringae* inoculation [92]. External application of xylanase in wheat resulted in higher resistance phenotype against *F. graminearum* and stimulated callose deposition in wheat CW. In soybean (*Glycine max*), overexpression of *xyloglucan endotransglycosylase/hydrolase* (*GmXTH43*) decreased the relative length of xyloglucan (XyG) chains in CW, impaired the ability of the CW to expand, and, consequently, limited the ability of parasitic nematode *H*. *glycines* to develop the feeding structure and thereby affected its parasitism [93]. 

Hemicelluloses can be acetylated similar to pectins. At least two protein families, the reduced wall acetylation (RWA) and trichome birefringence-like (TBL) families, are involved in the *O*-acetylation of hemicellulose [72,94,95]. Four *RWA* genes (*RWA1*–*RWA4*) are involved in the acetylation of xylan during secondary wall biosynthesis. Interestingly, the Arabidopsis *rwa2* mutant that contained 20% less polysaccharide O-acetylation was more resistant than wild type plants to *B. cinerea* [72], and the resistance was not linked to either the JA or ET pathway. The Arabidopsis mutant *powdery mildew resistant5* (*pmr5*), impaired in *TBL44*, was more resistant to *C. higginsianum*, whereas its resistance to *P. syringae* was on par with wild type [54,96,97]. 

In another study, rice double deletion mutant *tbl1tbl2* exhibited dwarf phenotype, reduced xylan acetylation, and reduced resistance to leaf blight disease [98]. A recent study showed that overexpression of *AtTBL37* in Arabidopsis results in increased acetylation polysaccharides, thickened secondary CW, and enhanced resistance against insect larvae *Spodoptera exigua* [99]. *AtTBL37* is activated by transcription factor *MYC2*, a central regulator in the JA signaling pathway. In a study with another TBL member, *Eskimo1* (*ESK1*) showed that the alterations in the CW acetylation in *esk-1* mutants were minor. However, these minor CWI impairments were sensed and triggered higher defense responses, and, also, the *esk1* plants displayed high freezing, drought, and salinity resistance [39,100,101,102], which shows that some of the biotic and abiotic resistance pathways are inter-linked. An interesting observation is that, despite the similarities of the constitutively activated defense responses of *esk1-7* and *irx1-6* mutants, the *esk1-7* plants were highly resistant to *P. cucumerina* but not to *H. arabidopsidis*, whereas *irx1-6* plants exhibited higher resistance to both the pathogens [38,39]. 

The methyl, acetyl, and feruloyl functional groups are believed to protect polysaccharides from the action of specific glycosyl hydrolases and to cross-link CW constituents for controlling cell extensibility [67,103,104]. Some microbes secrete CWDEs to specifically remove these functional groups to alter the CWI for their establishment. A recent study showed that apoplastic overexpression of an *A. niger acetyl xylan esterase1* (*AnAXE1*) in Arabidopsis reduced the CW xylan acetylation. Further, it was reported that the deacetylated xylans were easily digested by a β-1,4-endoxylanase and later easily extracted by hot water, acids, or alkali. However, the transgenic plants became highly resistant to the biotroph *Hyaloperonospora arabidopsidis* [105] as a result of released DAMPs from the deacetylated/hydrolyzed CW that constitutively triggered the immune system. Similarly, in another study, apoplast expression of *AnAXE1* in Arabidopsis and *B. distachyon* resulted in xylan deacetylation, priming of defense pathways, and higher resistance to necrotrophic fungi [36]. 

Some phenolic compounds, such as ferulate (FA), have key effects on CW structure, make CW recalcitrant to degradation, and function in plant defense against pests. FA dimers crosslink hemicellulosic polymers with arabinoxylan via an ester linkage or with lignin through an ether link [106]. Evidence shows that FA affects plant–pathogen interactions and that phenolic compounds are often induced in response to biotic stresses. FA is thought to play a role in fungal resistance and to be an important insect deterrent [107,108,109,110,111,112]. 

Apoplastic overexpression of an *A. niger ferulic* acid esterase (*AnFAE*) in tall fescue (*Schedonorus arundinaceus*) reduced the levels of CW ferulates and diferulates and increased the susceptibility to fall armyworms (*Spodoptera frugiperda*) [108]. Similarly, Arabidopsis and *Brachypodium* plants expressing *AnFAE* displayed a significant reduction in FA, decreased amounts of wall-associated extensins, and increased susceptibility to fungal pathogens in spite of increased expression of several defense-related genes [106]. In a recent study, Arabidopsis plants co-overexpressing two fungal acetylesterases (*AnAXE* and *AnRAE*) and a fungal feruloylesterase (*AnFAE*) showed an additive effect on defense priming and resistance to *B. cinerea* [35]. The obtained results provided evidence that combinatorial co-expression of some CWDEs can represent a useful technology for crop protection [35].

Impairment of Arabidopsis *WAT1* (*Walls Are Thin1*), a gene required for secondary CW deposition, conferred broad-spectrum resistance to pathogens, such as vascular bacteria *R. solanacearum* and *Xanthomonas campestris* pv. *campestris*, the soil-borne vascular fungi, *Verticillium dahlia*, and *Verticillium alboatrum*, and necrotrophic fungus *P. cucumerina* [113]. A severe reduction in the secondary wall thickness of fiber was noticed in the Arabidopsis *wat1* mutant. The resistance phenotype was due to higher SA accumulation and general repression of indole metabolism (including Trp) in *wat1* roots [113].

### 2.4. CW Callose Integrity and Plant Immunity

Callose is synthesized at the plasma membrane by callose synthases (CalS) or Glucan synthase-like (GSL) enzymes. In Arabidopsis, there are 12 GSL gene family members that fall into two groups: (i) fertility and cell division (GSL1, GSL2, GSL6, GSL8, GSL10) and (ii) structural CW reinforcement (GSL5, GSL7, GSL12) [114]. 

Arabidopsis mutant *gsl5* (*GSL5*, also known as *Powdery Mildew Resistant 4, PMR4*) does not deposit callose at fungal penetration sites. However, the mutant was highly resistant to *Golovinomyces cichoracearum* and *Golovinomyces orontii*, which was opposite to the expected result [115,116]. Additional studies revealed that the altered CW in the mutants resulted in the overexpression of SA pathway genes, which in turn conferred higher resistance [116]. However, the Arabidopsis *35S::GSL5* overexpressor plants accumulated high callose deposits at the fungal penetration site [117]. Interestingly, the overexpressor plants were completely resistant to *G. cichoracearum* and *B. graminis* without triggering JA or SA pathways. These findings indicate that GSL5-dependent callose deposition has complex roles in plant defense against pathogen invasion. A recent study reported that silencing of *HvGSL6* (functional orthologue of *AtGSL5*) in barley resulted in less callose accumulation and more susceptibility to *B. graminis* in comparison to the wild type [118]. 

Molecular and genetic evidence indicates that Class I b-1,3-glucanase (GLUI), which degrades callose, has an important role in callose turnover and regulation. In tobacco, silencing of *GLUI* results in increased callose accumulation at plasmodesmata, reduced the plasmodesmata size, and thereby restricted the viral movement and reduced the susceptibility to virus infection [119]. The transgenic plants reduced and delayed the cell-to-cell spread of Tobacco mosaic virus, Potato virus X, and Cucumber mosaic virus. Similarly, the Arabidopsis *atbg_pap* mutants, which lack a plasmodesmata associated b-1,3-glucanase (AtBG_pap), exhibited an increase in callose accumulation and reduction in Tobacco mosaic virus spread [120]. 

### 2.5. CW Lignin Integrity and Plant Immunity

Lignin is an aromatic polymer mainly deposited in secondary CWs, providing strength and rigidity. In monocot and dicot plants, lignin is mainly composed by the monolignols coniferyl and sinapyl alcohol that give rise to the guaiacyl (G) and syringyl (S) units in the lignin polymer, respectively. p-Coumaryl alcohol, forming the p-hydroxyphenyl (H) units, is a minor monolignol slightly more abundant in monocot than in dicot CWs [121]. The biosynthesis and deposition of lignin in secondary CWs are developmentally programmed. Moreover, lignin phenolic polymers are synthesized and deposited in CWs to act as a physical barrier in response to biotic and abiotic stresses and CW perturbations [122,123]. 

Evidence for the role of lignin and soluble phenolics in plant defense has been obtained from the overexpressor transgenic plants and mutants with contrasting lignin amounts or compositions [121]. For example, transgenic tobacco plants, constitutively overexpressing *phenylalanine ammonia lyase* (*PAL*) genes, conferred higher tolerance towards *Cercospora nicotianae* and *Phytophthora parasitica* pv. *nicotianae* [124,125,126]. Tobacco plants overexpressing *L-PAL* produced high levels of chlorogenic acid and exhibited markedly reduced susceptibility to fungal pathogen *C. nicotianae*, although their resistance to tobacco mosaic virus is unchanged, and the resistance is linked to SA [126]. 

The rice mutant for *snl6* gene (a suppressor of negative regulator of *PR* genes) had a lower lignin content and reduced resistance to the bacterium *Xanthomonas oryzae* pv. *oryzae* [127]. The results indicate that *SNL6* has dual roles in the resistance response, such as activation of PR defense genes of the SA pathway and lignin biosynthesis. Previous studies have shown that expression of the *PAL1* gene is required for biosynthesis of secondary metabolites, including lignin and SA. Rice plants lacking *BSR-K1*, a gene required for modulating turnover of the *PAL1* gene, had elevated levels of *OsPAL1* mRNA and SA. This resulted in enhanced lignin content, which conferred broad-spectrum resistance to fungus *Magnaporthe oryzae* (rice blast) and bacterium *X. oryzae* (leaf blight) [128].

Lignin content is normally positively correlated with plant pathogen resistance. Overexpression of a rice transcription factor, *OsMYB30*, was found to activate the expression of lignin biosynthesis-associated genes *Os4CL3* and *Os4CL5*. It resulted in higher accumulation of lignin subunits (G and S) and FA, strengthened sclerenchyma cells and ROS burst, and overall resulted in higher resistance to *M. oryzae* penetration [129]. However, there is also a scenario in which lignin content was negatively correlated to disease resistance. In alfalfa (*Medicago sativa*), down-regulation of the shikimate/quinate *hydroxycinnamoyl transferase* (*HCT*) gene resulted in plants with reduced lignin content but, interestingly, resulted in enhanced tolerance to fungal pathogen *Colletotrichum trifolii*. This activation of defense responses was hypothesized to be triggered by bioactive pectin DAMPs released from the loose secondary CW [130]. Consequently, it increased the levels of SA, JA, and ABA, the levels of pathogenesis, and abiotic stress-related genes, which resulted in enhanced tolerance to fungal infection and drought [130]. 

In cotton, transcription factor *MYB4* is known as a negative regulator of lignin biosynthesis. Expression of *MYB4* in Arabidopsis resulted in reduced lignin production, reduced CW recalcitrance, oligogalacturonides release, activation of JA biosynthesis and defense responses, and, consequently, enhanced disease resistance against *Verticillium dahliae* [131]. In cotton (*G. hirsutum*), overexpression of cotton *DIRIGENT1* gene enhanced the lignification and reduced the spread of *V. dahliae* [132].

Analysis of Arabidopsis mutants defective in lignin biosynthesis and of transgenic plants overexpressing lignin biosynthesis genes has vastly contributed to unravelling lignin’s role in plant immunity. For example, a *pal1/2/3/4* quadruple mutant having only 25% residual CW lignin content and 25% residual SA level exhibited dwarf phenotype and was hypersusceptible to *P. syringae* [133]. The Arabidopsis *comt* mutant (COMT; caffeate *O*-methyltransferase, involved in lignin biosynthesis) had less lignin and JA level and became more susceptible to *P. syringae pv. tomato DC3000*, *B. cinerea*, *A. brassicicola* and *X. campestris pv. campestris*, and *B. graminis f. sp. Hordei* [134]. Arabidopsis *f5h1* mutant (*sinapate-deficient* mutant) exhibited higher susceptibility to fungal pathogen *S. sclerotiorum* and vascular fungus *Verticillium longisporum* [135]. The lignin content in *f5h1* mutants was similar to the wild type, but the lignin lacked S units and sinapate esters (the inhibitor of fungal growth) [135].

### 2.6. Expansins Reinforce CW Resistance to Stresses

Expansins are a family of plant and microbial proteins present in the CW that plays an essential role in CW remodeling through their binding capacity to cellulose and other CW polysaccharides. They also play roles in non-catalytic disruptive activity aimed to create weak bonding between polysaccharides (loosening of CW) in CW extension. Expansins are also induced under several stress conditions [103,136]. Expansins are classified as expansin-like type a and type b (subfamilies EXLA and EXLB). Subfamily X (EXLX) includes microbial expansins, from bacteria, fungi, and oomycetes, with structural similarity to β-expansins [137,138]. Few studies were carried out to elucidate the involvement of expansins in CWI-mediated plant defense. 

Ectopic overexpression of an *Arachis duranensis AdEXLB8* in tobacco resulted in tolerance to drought and resistance to white mold causative necrotrophic fungus, *Sclerotinia sclerotiorum*, and to the nematode *M. incognita* [139]. Overexpression changed the CW nano-biomechanical properties of CW and activated JA and ABA signaling pathways that resulted in resistance phenotype [139]. This result is in line with the earlier finding that overexpression of *AdEXLB8* resulted in a significant decrease in the number of galls produced in the soybean (*Glycine max*) and peanut (*Arachis hypogaea*) hairy roots by nematodes, *Meloidogyne javanica* and *M*. *arenaria*, respectively [140]. Contrarily, upon mutation of another Arabidopsis *expansin-like A2* (*EXLA2*) gene, the plants became resistant to *B. cinerea* and *Alternaria brassicicola* [141]. The results also showed that *EXLA2* expression is not modulated by SA or JA-ET signaling pathways but by ABA. 

Expansins are also inherited by some phytopathogenic bacteria, and evidence indicates that they act as virulence factors for infecting the host plants. The studies with *exl1* mutant strain of *Pectobacterium brasiliense* and *Pectobacterium atrosepticum* revealed that EXL1 acts on the plant tissue, probably by remodeling a CW component or altering the barrier properties of the CW. However, inoculation of mutant bacterium induced the plant defense response, which resulted in ROS burst and induction of marker genes of the JA, ET, and SA signaling pathways in Arabidopsis and eventually resulted in resistance reaction against *P. brasiliense* and *B. cinerea* [142].

### 2.7. CW Hydroxyproline-Rich Glycoproteins and Plant Immunity

Plant CW Hydroxyproline-rich glycoprotein (HRGP) superfamily proteins consist of three families, extensins, arabinogalactan proteins (AGPs), and proline-rich proteins [143]. Extensins and AGPs are most abundantly present. Many recent studies are devoted to examining the biological roles of AGPs in a wide range of plant processes. However, little attention has been given to their role in plant–microbe interactions. Few of the recent studies showed that HRGPs are indeed involved in host–pathogen interactions or wounding responses [143]. 

Extensins have a continuous sequence of hydroxyproline residues (known as extensin motif). Extensins have been described to fortify the plant CW through cross-linking, thus providing enhanced mechanical protection against pathogen invasion. The cross-linking requires glycosylation of serine residue with galactoses and hydroxyproline residues with oligoarabinoses. 

Several lines of evidence have suggested that extensins are key elements in root protection against soil-borne pathogens. In Arabidopsis, an extensin gene, *AtEXT1,* is mostly expressed in root tissues. Interestingly, overexpression of *AtEXT1* in leaves limited the spread of *Pseudomonas syringae* DC3000 infection and symptoms. Basal defenses and signal transduction pathways involved in plant defense were not perturbed in transgenic plants [144]. However, immunofluorescence imaging using specific monoclonal antibodies (mAbs; i.e., LM1, JIM11, and JIM20) revealed that both elicitor and infection by pathogen-induced reorganization of extensin epitopes occurs within the cell wall [145,146,147]. A recent study using Arabidopsis mutants (impaired in extensin arabinosylation) revealed the importance of glycosylation in limiting the invasion of root cells by the pathogen [148]. 

AGPs are cell-wall-localized glycoproteins, often GPI-anchored, which participate in root functions at many levels. They are involved in cell expansion and differentiation, regulation of root growth, interactions with other organisms, and environmental response. Due to the complexity of cell wall functional and regulatory networks, despite a large amount of experimental data, the exact molecular mechanisms of AGP action are still largely unknown.

However, there is substantial evidence of occurrence of compositional changes of AGPs in roots or root exudates in response to pathogens or parasites. In *Solanum tuberosum*, AGPs (detected with LM2 and JIM15 antibodies) were upregulated in root exudates in response to elicitors derived from *Pectobacterium atrosepticum*, the soft rot pathogen of potato [149]. In *Musa* spp. roots, AGPs were upregulated by *Fusarium oxysporum* f. sp. *cubense* infection [147]. Higher levels of AGPs and EXTs were detected in the roots of a *Benincasa hispida* cultivar, which is resistant to *F. oxysporum* f. sp. *benincaseae* [145]. 

Few studies revealed the role of individual AGPs by modulating AGP genes expression. Arabidopsis *rat1/agp17* (*resistant to Agrobacterium transformation 1*) mutant, defective in AtAGP17 protein, is resistant to Agrobacterium transformations of root segments [150,151]. Two other AGPs, AtAGP12 and AtAGP24, were induced in the roots of Arabidopsis upon infection with *P. cucumerina*. Overexpression of *AtAGP24* increased the host susceptibility to *P. cucumerina*, which is evidence for its involvement in the pathogen response [152]. Knocking out of *AtAGP8* gene in Arabidopsis resulted in increased host susceptibility to root-knot nematode *M. incognita* [153]. The susceptibility is related to the altered CW composition. The mutant CWs had high levels of methyl-esterified homogalacturonans, xyloglucans, and arabinans, allowing for plasticity and cell expansion, which favored nematode infection [153]. 

### 2.8. Role of Lytic Polysaccharide Monooxygenases and Cuticle in Plant Immunity

A novel family of CWDEs, the lytic polysaccharide monooxygenases (LPMOs), were found to play a role as a pathogenicity factor in pathogens by oxidatively degrading polysaccharides. Further, the oxidized oligosaccharides produced by the LPMOs do not trigger host defenses/plant immunity, which favors the pathogen’s penetration and survival. For example, silencing an HG-degrading LPMO in the oomycete *Phytophthora infestans* resulted in its reduced virulence in potato [154]. The oxidized oligogalacturonides (OGs) generated by this enzyme from the HG fragments are unable to trigger host defenses compared to non-oxidized OGs [155]. Similarly, chito-oligosaccharides, produced by an LPMO in the haustoria of powdery mildew pathogen *Podosphaera xanthii,* were found to suppress the chitin-induced plant immunity in melon [156].

On the contrary, overexpressing an Arabidopsis LPMO gene, *AtOGOX1* (*oligogalacturunoids oxidase 1*), increased Arabidopsis resistance to *B. cinerea* despite the reduced induction of the defense genes. The data suggested that the increase in resistance exhibited by the *OGOX1* overexpressing plants is not due to the triggered defense responses but due to the fact that the plant tissue became more recalcitrant to degradation and also due to dampened counterproductive hyperactivity of OGs [155]. Similarly, an LPMO-oxidized cellulose oligosaccharides treatment triggered immunity in Arabidopsis, conferring resistance to *B. cinerea* [157]. The study conferred that cellulose oligosaccharides increased ET, JA, and SA levels, callose deposition, and camalexin biosysnthesis. Evidence showed that the oxidation of OGs and cellodextrins (CDs) inactivated their DAMP activity and also made them a less palatable food source for microbial pathogens [158]. Collectively, the experimental evidence reveals that LPMOs might play an important role in host plant–pathogen interactions.

In plants, the cuticle is the first line of defense against microbial invaders. This lipophilic layer comprises the polyester cutin embedded in cuticular wax, composed of a mixture of fatty acids modified with functional groups [159,160]. Several cuticle mutants and overexpression lines were employed to study the effect of modification of cuticle in plant defense priming and reaction against plant pathogens, and it has been elaborately reviewed recently [161,162,163].

## 3. Cell-Wall-Derived Oligosaccharides Trigger Plant Immunity

Plant cell wall fragments (DAMPs) generated by either CWMEs or CWDEs, released in the form of various oligosaccharides, serve as elicitors for plant defense responses. External application of natural CW-derived and synthetic oligosaccharides induces plant immunity [16,158]. Microbial CW-derived MAMPs and herbivore CW-derived HAMPs are known to induce defense signaling events. However, in this review, we focused mainly on the essential aspects and examples of research studies demonstrating the role of plant CW-derived oligosaccharides in triggering plant immunity. Several types of oligosaccharide fragments might be released during pathogenesis, and some could act as DAMPs. Well-known DAMPs are oligogalacturonides (OGs) released upon homogalacturonan degradation, followed by cellulose breakdown products, the cellodextrins (CDs). 

Cellodextrins (cellobiose, cellotriose, cellotetraose) generated from cellulose, especially cellotriose, activate plant immunity on a higher scale and induce disease resistance in Arabidopsis and grapevine. Cellodextrins exhibit a marked induction of cellular responses, such as oxidative burst, the elevation of free cytosolic calcium, activation of MAPK cascades, and the expression of PR genes and callose deposition [164,165,166,167]. 

Similarly, in Arabidopsis, tobacco, and grapevine, xyloglucan-derived oligosaccharides increased the resistance against pathogens by inducing innate immune responses. This response includes activation of mitogen-activated protein kinases (MPK 3 and 6), ROS burst, defense genes induction, callose deposition (*PMR4* gene), camalexin (*PAD3* gene) production, and activated salicylate (*PR1* gene), jasmonate, and ethylene (*PDF1.2* gene) pathways [168,169]. In grapes, the transcript level of two enzymes, phenylalanine ammonia lyase (*PAL*) and stilbene synthase (*STS*), genes responsible for the resveratrol phytoalexin biosynthesis, were increased. Recent work showed that arabinoxylan oligosaccharides, especially pentasaccharide (XA3XX), were highly active as DAMPs and triggered strong immune responses [170]. Mixed-linked glucans, which are widely present in Poaceae and also present in bryophytes and algae and fungal CW, were found to trigger a wide variety of immune responses [165,167,169,171,172,173,174]. 

The well-studied elicitors derived from plant cell wall are OGs (with different degrees of polymerization), produced by pectin degradation [175,176,177,178,179]. The degree of polymerization (DP) of OGs produced by fungal pectin lyases ranges from DP3 to 10, of which DP4 and DP5 are the main form. By contrast, the OGs derived from polygalacturonases are mainly DP2 and DP3. Short OGs (DP1-7) can also induce the expression of PR genes in potatoes and tomatoes despite being less effective in activating hormones than long OGs [177,179,180,181]. Plasma-membrane-associated protein wall-associated kinases (WAK) have been characterized as the receptor for recognizing OGs. Its N-terminal extracellular domain preferentially binds to the de-esterified pectin [182]. Arabidopsis encodes around 25 members of the WAK family, among which WAK1 and WAK2 contribute to disease resistance against various pathogens [183,184].

## 4. CWI Surveillance, Receptors, and Signaling Components 

Plants have well-established surveillance mechanisms to sense any changes in CW perturbations due to biotic/abiotic stresses through the plasma-membrane-located pattern-recognition receptors (PRR) [11,12]. Upon sensing any CW perturbations or DAMPs or MAMPs or HAMPs, it is well known that the plant cells activate plant defense responses. There is a sequence of events carried out by the cells, which includes signaling relay through protein kinases, Ca_2_+ influx, reactive oxygen species (ROS) production, protein kinases cascade activation, protein phosphorylation, transcriptional reprogramming, induction of defense-related genes, cell wall reinforcement, generation of anti-microbial secondary metabolites, enzymes to digest the microbial wall structures, and activation of the ET, SA, and JA pathways for amplifying and spreading defense responses to distal tissues [12,13] (Figure 1). 

In spite of considerable research, only a few molecular components of these CWI-related plant surveillance and signaling pathways for a specific cause have been identified to date, mainly due to high functional redundancy. Here, we summarized the recent research findings of specific host plant–pathogen surveillance receptors and defense pathway components only related to plant CW polysaccharides. In plants, the most described CWI-derived elicitor signaling pathways function through receptor-like kinase (RLKs), which is one of the largest gene families, with more than 600 members found in Arabidopsis. The most studied RLK subfamily in relation to CW signaling and pathogen defense is Catharanthus roseus Receptor-Like Kinase 1-Like (CrRLK1L) [185,186,187]. In Arabidopsis, this subfamily consists of seventeen members, and seven have been functionally characterized putatively [186]. One of the CWI-related CrRLK1L receptors, THESEUS1 (THE1), was involved in the signaling pathway that includes induction of callose and lignin synthesis, enrichment of homogalacturonan, involvement of JA, ET, and SA hormones [11,38,188,189,190,191]. 

Apart from THE1, another RLK, FERONIA (FER), is the most widely studied receptor [192]. FER also acts as a scaffold protein, recruiting a complex that includes the elongation factor thermo unstable receptor (EFR), flagellin sensing 2 (FLS2), and brassinosteroid-associated kinase 1 (BAK1) to initiate defense pathway signaling [193]. Arabidopsis *fer* mutants were resistant to a biotroph *Golovinomyces orontii*, which implies that FER negatively regulates plant immunity [194]. In the *fer* mutants, the *PDF1.2* marker gene transcript was induced, which shows that FER is involved in ET- and JA-mediated defense pathways [195]. Contrarily, *fer* mutants were susceptible to a biotroph, *H. arabidopsidis*, and a hemibiotroph, *C. higginsianum* [195,196]. It seems that FER functions both in positive or negative regulation of immune responses. Intriguingly, recent evidence indicates that FER binds pectins through its extracellular domains to monitor CW Integrity [197].

**Figure 3:**
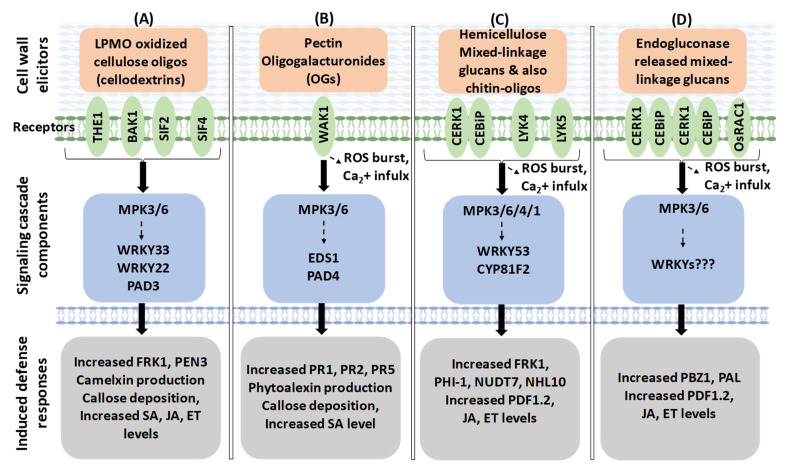
Defense pathways involved against the CWI alterations triggered by CWMEs or CWDEs. (**A**) Plant LPMO-oxidized cellulose oligosaccharides activated a signaling pathway, which resulted in resistance to *B. cinerea* in Arabidopsis. (**B**) Released CW pectin OGs activated an immune pathway that resulted in Arabidopsis resistance to *B. cinerea* and *P. syringae* pv. *tomato* DC3000. (**C**) Mixed-linked glucans (MLGs) DAMPs from digested rice CW triggered PTI, and, as a result, the Arabidopsis plants became resistant to *H. arabidopsidis*. (**D**) CW-MLGs released from pathogen endoglucanases-overexpressor rice plants activated immune responses, resulting in enhanced resistance to *M. oryzae*.

A recent study showed that plant LPMO-oxidized cellulose oligosaccharides’ treatment enhanced disease resistance to *B. cinerea* in Arabidopsis [157]. The study suggested that two plasma-membrane-localized RLKs, BAK1, and THE1 might form a complex with two LRR-RLK receptors, stress induced factor 2 and 4 (SIF2, SIF4) for the perception of cellulose oligosaccharide DAMPs. Further, it showed that camalexin biosynthetic pathway component genes, such as *MPK3/6*, *WRKY22*, *WRKY33*, *PAD3*, and *PEN3,* were induced and resulted in accumulation of camalexin (Figure 3). Increased levels of callose deposition, also SA, JA, ET hormones, were noticed in Arabidopsis. The study also confirmed that cellulose-oligosaccharides-induced camalexin accumulation happens independently of MPK3/6 phosphorylation. 

Apart from RLKs, other types of kinases involved in surveillance of the CWI are wall-associated kinases (WAKs), which are the only receptors experimentally validated at present as a CWI receptor for binding to pectin and OGs [182,198]. The Arabidopsis genome has five WAKs and 21 WAK-like genes (WAKL) [199]. WAKL genes are also reported to be involved in immune responses in wheat, maize, and rice [200,201]. WAK1 mainly binds to non- or low-methyl esterified OGs, and the arginine and lysine residues located at the N-terminus of the extracellular portion of the receptor contribute to the binding. Recent studies identified WAK-mediated signaling pathway components that include production of ROS and NO, involvement of MAPK6 and transcription factors *EDS1* (*enhanced disease susceptibility1*) and *PAD4* (*phytoalexin deficient4*), accumulation of phytoalexins and SA callose deposition, and expression of defense genes *PR1*, *PR2*, and *PR5* [202,203] (Figure 3). In total, the WAK-mediated surveillance signaling and activation of immunity resulted in Arabidopsis resistance to *B. cinerea* [158] and *P. syringae* pv. *tomato* DC3000 [204]. 

Another PRR subfamily is lysin motif domain proteins, which includes the chitin-elicitor receptor kinase1-chitin elicitor binding protein (CERK1-CEBiP) receptor complex, LYM, and LYK proteins. In rice, the chitin-elicitor receptor kinase 1-chitin-elicitor binding protein (CERK1-CEBiP) receptor complex was required for recognizing the chitin oligosaccharides (from fungal pathogen CW) [205,206]. OsCEBiP directly binds to chitin oligomers by its lysin motif (LYM) domain and forms a receptor complex with OsCERK1 to induce chitin signaling [207,208]. Another main component of fungal CW is non-branched β-1,3-glucans oligosaccharides. These oligosaccharides from *P. cucumerina* were reported to act as an MAMP, bind with AtCERK1-CEBiP receptors, and trigger immune responses in Arabidopsis, such as elevation in cytoplasmic calcium, ROS, phosphorylation of MAPKs, and upregulation of PTI marker genes (*CYP81F2*, *WRKY53*, *FRK1*, *PHI-1*, and *NHL10*) [173]. However, AtCERK1 only functions as a co-receptor because it does not directly bind to oligosaccharides such as 1,3-β-(Glc)6 [172,173]. Recently, MLG trisaccharides and tetrasaccharides derived from the rice cell wall have been characterized as novel DAMPs perceived by AtCERK1-LYK4-LYK5 complex and triggered PTI components, as mentioned above, and the MLG-treated Arabidopsis plants became resistant to *H. arabidopsidis* [171] (Figure 3). 

Recently, in rice, the endoglucanases secreted by *M. oryzae* that targets hemicellulose of the rice CW was reported to release MLG DAMPs (trisaccharides and tetrasaccharides). The released DAMPs were perceived by OsCERK1-CEBiP receptors and activated immune responses resulting in plant resistance. The plant immune responses noticed were: MLG DAMPs induced OsCERK1-OsCEBiP dimerization, recruitment of OsRAC1 GTPase, increased ROS burst, MAPK activation, and PTI marker genes (*PBZ1 and PAL*) expression [209] (Figure 3). Overexpression of two *M. oryzae* endoglucanases (*MoCel12A* and *MoCel12B*) in rice resulted in enhanced defense response and enhanced resistance. As expected, *oscerk1* and *oscebip* mutant rice plants showed opposite results. 

The heptamaloxyloglucan (DP = 7) oligosaccharide obtained from apple pomace acted as an elicitor (through a still unknown membrane receptor) in grapevine and Arabidopsis [169]. This oligoxyloglucan induced fast dose-dependent phosphorylation of MAPKs within the first 10 min of treatment. Late responses reported were induction of the SA and camalexin pathways in Arabidopsis and *PAL* and *STS* genes of the resveratrol biosynthesis pathway in grapevine [169]. Additionally, CW reinforcement with callose deposition was reported as part of the responses induced by oligoxyloglucan [169,210].

Recent work demonstrated that an Arabidopsis mutant impaired in *Arabidopsis Response Regulator 6* (*arr6*) enhanced disease resistance to *S. sclerotiorum* and *P. syringae* pv. *tomato* DC3000 by accumulating DAMPs, mainly an arabinoxylan pentasaccharide (XA3XX). Treatment of extracted arabinoxylan oligosaccharide not only enhanced resistance to the pathogens but also induced full PTI defense responses, such as Ca2+ influx, ROS burst, and induced MAPKs phosphorylation and induction of five PTI-marker genes (*CYP81F2*, *WRKY53*, *PHI1*, *FRK1*, and *NHL10*) [170].

## 5. Conclusions and Perspectives

The plant CW is not just a passive plain physical barrier. A CW is a highly dynamic and complex structure consisting of networks of molecules that constantly change during growth and development. Plant cells constantly monitor the status of the CW with various types of receptors located at the plasma membrane for any alterations that occur during normal growth or any biotic/abiotic stresses and dynamically respond. To date, various studies identified numerous processes involved in CW alterations and their effect on CWI and signaling pathways, the analysis of which has greatly contributed to a better understanding of the molecular mechanisms behind plant immunity.

In this review, we focused on presenting mainly the studies related to CW alterations through mutations or expression of plant endogenous CWMEs and microbial CWDEs, priming of signaling cascades, and their impact on disease resistance phenotypes. There are numerous CWMEs, and CWDEs specifically alter CW molecules. The fine structural remodeling of particular CW polysaccharides via side chain cleavage, de-esterification, and partial depolymerization/degradation leads to initiation of specific signaling pathways related to these particular modifications. Studies showed that most of the evidence about CWI signaling in plants comes from mutant studies, where biosynthesis of various CW components is compromised, causing wall structural damage or rearrangement.

Most research in this area has been guided by a reductionist approach; however, a full picture of plant immunity, including connectivity of different immune pathways, is not fully elucidated. It might be due to the high functional redundancy of pathway components observed with the existing materials and methods used in current research. An interesting observation is that defense priming due to altered CWI will not always result in a resistance outcome. Sometimes, even the susceptible phenotype had a fully activated immune system. Moreover, some CWI alterations result in opposite phenotypes to two different pathogens or no change in phenotype. Overall, the information currently available is highly limited to clearly ascertain the full picture of plant immunity, which demands a systematic approach. Finding out the intricate molecular mechanisms for each specific plant–pathogen interaction and the dynamic nature of whole plant immunity for all the biotic interactions of a specific host plant is essential to successfully address the problems arising by these biotic or abiotic stresses.

Post-synthetic modification of CW components via their partial remodeling by overexpressed CWMEs/CWDEs and their inhibitors is an emerging and powerful method to specifically modify and investigate CWI signaling mechanisms behind the specific change. This approach, in addition to other methods, such as gene mutations and using CW derived/synthetic oligosaccharides, can assist in revealing specific components of signaling pathways initiated in response to such highly specific cell wall modifications. This could allow us to dissect otherwise highly complex cell responses that occur during plant development or defense responses. The information that is generated would be highly useful to engineer new strategies in crop protection, the bioenergy/biofuel sector, and feedstock improvement, and, overall, will significantly contribute to improvements in crop productivity and sustainability.

## Data Availability

Not applicable.
